# Fast surface reconstruction of human brain MRI: benchmarking deep-learning based morphometry tools

**DOI:** 10.1038/s41598-026-55397-w

**Published:** 2026-06-12

**Authors:** Victor B. B. Mello, Richard McKinley, Roland Wiest, Christian Rummel

**Affiliations:** 1 Support Center for Advanced Neuroimaging (SCAN), University Institute of Diagnostic and Interventional Neuroradiology University of Bern, Inselspital, Bern University Hospital, Bern, Switzerland; 2 European Campus Rottal-Inn, Technische Hochschule Deggendorf, Max-Breiherr-Straße 32, 84347 Pfarrkirchen, Germany

**Keywords:** Neuroimaging, Quantitative image analysis, Cortical atrophy, Computational biology and bioinformatics, Engineering, Health care, Mathematics and computing, Medical research

## Abstract

Time efficient and reliable pipelines for quantitative evaluation of structural brain MRI are essential to utilize the potential of morphometry tools for large scale research projects as well as to pave the path towards future clinical applications. In our work, we have explored this idea by evaluating three deep learning models for brain segmentation and cortex parcellation (DeepSCAN, FastSurferCNN and QuickNAT) as input for an 11-min surface reconstruction pipeline adapted from the well studied open source software package FreeSurfer. Performance was assessed using both, large publicly available human MRI datasets and a synthetic dataset with known metrics and reference surfaces. Evaluation criteria included closeness to the surface reconstruction by FreeSurfer’s full recon-all pipeline, reproducibility within same-session rescans, performance stability across a wide age range, sensitivity to variations of the grey-white contrast in the MRI and accuracy regarding metrics of synthetic surfaces. Metrics derived from the DeepSCAN-based pipeline demonstrated the highest agreement with FreeSurfer in the human data and the greatest fidelity to the expected metrics in the synthetic dataset. Our findings identify the DeepSCAN-based surface reconstruction pipeline as a rapid, yet reliable alternative to established research-grade structural MRI processing. Time expenditure and reliability suggest it is suitable for research applications with high-throughput requirements. This is an essential first step towards necessary subsequent studies aimed at evaluating robustness, pathological variability, and utility in the context of clinical diagnostics.

## Introduction

Quantitative analysis of structural Magnetic Resonance Imaging (MRI) has been available for more than two decades^1–3^, enabling detailed investigations of the healthy brain and different neurological disorders across the human lifespan^4^. Whole-brain reconstruction from a structural MRI is typically performed by time consuming processing with one of a handful of well explored tools: voxel-based morphometry (VBM), as provided by SPM^5^, ANTs^6^, and FSL^7^, or cortical surface reconstruction for surface-based morphometry (SBM), as performed by Freesurfer^8^, BrainSuite^9^ and CIVET^10^. The anatomical complexity of the human cortex, combined with substantial inter-individual variability and high data dimensionality, renders these reconstructions both computationally-intensive and time-consuming.

From a clinical standpoint, structural MRI enables a non-invasive visual assessment of the brain anatomy. It is an integral component of routine neuro-diagnostics, supported by well-established protocols for diagnosing and monitoring a broad spectrum of neurological disorders^11–13^. In parallel, various forms of brain morphometry derived from structural MRI have significantly contributed to a deeper understanding of healthy brain development and aging, but also disease manifestation and in some cases even disease mechanisms. Despite its widespread use in research, the incorporation of advanced morphometric analyses into clinical workflows^14–17^ remains limited due to the computational demands and complexity of existing reconstruction pipelines. First commercial applications of brain morphometry have become available that are cleared for clinical use. Their limitations are threefold: (i) they mainly focus on grey matter (GM) volume, (ii) they lack regional specificity by focussing on the lobar level and (iii) their scientific and clinical validation still requires expansion^18,19^. As a first step towards bridging the gap between research and clinical practice, it is essential to devise and validate new pipelines that are not only capable of fully reconstructing the cortical surface of a structural MRI in a time efficient manner, but also are accurate and robust against the inherent variability in the reality of clinical applications. They must be robust against using data from multiple MRI scanner models and vendors, different image contrast between grey matter and white matter and different subjects.

Deep-learning (DL) and particularly convolutional neural network (CNN)^20^ based methods have become the first choice for fast whole-brain segmentation and parcellation. In the last few years, the field has rapidly evolved from architectures that generate brain segmentation with a runtime of 15–60 min^21–26^ to models with a runtime of less than a minute^27–30^. However, much progress is still to be made in the use of DL for cortical surface reconstruction. The few models available^31–33^ offer significant speed advantages (less than a 1 min runtime) but often suffer from topological inconsistencies, such as self-intersecting surfaces or anatomically implausible geometries. While recent advances^33^ have improved surface regularity, reducing self-intersections from about 1.5% of the faces on the reconstructed pial surface by a factor of six, these methods still do not guarantee a topologically valid reconstruction. This makes FastSurfer^30^ the currently fastest whole-brain surface reconstruction pipeline described in the literature, that guarantees topologically correct surfaces. FastSurfer reconstructs a cortical surface in about 90 min by combining DL-based brain segmentation with parts of the established FreeSurfer pipeline.

Novel structural MRI reconstruction techniques require extensive validation to probe their accuracy, reliability and robustness. A major obstacle to such evaluation is the lack of datasets with indisputable ground-truth reconstruction. The manual delineation of such gold standard data by human experts^34^ is a time-consuming task that requires highly specialized anatomical knowledge and may present significant variability across different experts^35^. As an alternative, many studies compare to FreeSurfer outputs as commonly accepted silver standard, or use repeated MRI acquisitions to assess scan-rescan reliability. Both approaches have their own limitations, the former leading to an evaluation bias^36^ and the latter being only an indirect indication of reconstruction accuracy. A third approach is the use of synthetic datasets generated with a controlled deformations on a real brain MRI. As cortical thickness is a popular metric used in the study of neurodegenerative and neurological conditions, synthetic phantom data typically focus on inducing atrophy by computationally shrinking the cortex. By producing a topology-preserving deformation field from the pial surface to the WM-GM interface, the synthetic atrophy is generated by moving the pial surface along this field, in the direction of the WM. The available models to create a synthetic cortical atrophy can be based on the Jacobian of the transformation^37–39^, biomechanical models^40–42^, binary morphological operations^43^ or DL^44,45^. Although such datasets do not constitute anatomical ground truth, they provide a controlled reference, here referred to as synthetic ground-truth, which enables systematic evaluation under known conditions.

Aiming to extend the analysis of the relative performance of DL-based segmentation tools beyond the Dice similarity coefficient between the alternative cortical parcellation and a silver standard reference provided by Freesurfer, in this work we examine also the accuracy of morphometric variables extracted from these segmentations like the cortical surface area, GM volume and thickness. To enable future applications in the clinical context, where runtime will be an issue, we have developed a fast surface reconstruction pipeline that was heavily inspired by the approach presented in^30^. Our solution yields a reliable surface reconstruction pipeline that can be applied to any high-quality segmentation method, reducing processing time for whole-brain reconstruction down to minutes. In this work we evaluate three state-of-the-art DL-based tools for whole-brain MRI segmentation—DeepSCAN^29^, FastsurferCNN^30^ and QuickNAT^28^—for surface based brain morphometry. To prove the concept, we have performed a direct comparison between the morphological variables obtained from DL-based segmentations by our proposed fast surface reconstruction pipeline and FreeSurfer for a wide variety of subjects and MRI sequences. Finally, using the synthetic dataset generated by^45^, we have benchmarked each one of the three alternative reconstruction pipelines based on the similarity to the synthetic ground-truth surface and reproduction of the expected surface-based metrics.

## Materials and methods

### Imaging data

We study morphometric pipelines applied to both, real T1w human brain MRI (obtained from open-access databases) and synthetic data (the DL-based cortical atrophy phantom generated by^45^ and obtained from the original authors upon direct request). The human dataset aims to provide a proof of concept for the fast surface reconstruction pipeline using DL-based whole-brain segmentation. In order to demonstrate the generalizability of the fast surface methodology, the human dataset was compiled with a high variability regarding age, pathologies and MRI scanning parameters for both male and female subjects. The dataset combines: (i) a random sample of 1000 young subjects from the Adolescent Brain Cognitive Development (ABCD) study^46^; The healthy young adults from the Human Connectome Project (HCP)^47^; (iii) The Phantom of Bern (PoB)^48^, a longitudinal experiment with two healthy adults in which the MRI sequence parameters inversion time (TI) and repetition time (TR) are systematically changed to control the contrast between the WM and GM signal intensities; (iv) The elderly subjects, both healthy and with diverse degrees of cognitive impairment, from the OASIS3 dataset^49^. A summary description of the human MRI dataset is given in Table 1. Both, the HCP and the OASIS3 dataset contain subjects with more than one MRI scan acquired during the same session, allowing a scan-rescan reproducibility analysis. With the exception of the PoB, which was designed for a controlled change on the MRI scanning parameters, all datasets were obtained in studies with more than one scanner and distinct acquisition parameters.

**Table 1:** Summary of the human MRI dataset. CTL, healthy control, AD, Alzheimer’s disease.

Datasets	Diagnosis	N subjects		N scans		Age range [years]	Scanners
		Male	Female	Male	Female		
ABCD	CTL	500	500	500	500	9–10	3
HCP	CTL	507	606	815	968	26–35	3
OASIS3	CTL	299	438	777	1226	42–97	3
OASIS3	AD	193	167	338	298	50–95	3
PoB	CTL	2	0	18	0	30’s and 40’s	1

As described in the original paper^45^, the synthetic dataset was generated using 20 heathy controls from the Alzheimer Disease Neuroimaging Initiative (ADNI)^50,51^ by exploiting the inherent one-to-one correspondence between vertices of the WM surface and vertices of the pial surface on FreeSurfer’s reconstructed meshes. A uniform controlled synthetic atrophy across the GM was introduced moving the pial surface in the direction of the WM with a fixed distance. Then, based on these deformed meshes, a synthetic MRI was generated using a generative adversarial network (GAN) model^52^. This procedure was designed to assess sensitivity to controlled, sub-voxel changes in cortical thickness, not to provide an anatomically accurate ground-truth surface. Nevertheless, as the deformed meshes directly underlie the generation of the synthetic MRI data, they serve as a synthetic ground-truth, providing a controlled reference for comparison. Consequently, the synthetic dataset provides an expected value for the cortical thickness and synthetic ground-truth surfaces for the pial and white matter, allowing a direct comparison to benchmark each reconstruction methodology. The values of atrophies provided by the authors range from zero (no modification of the surfaces) to 1 mm.

### Processing pipeline with different segmentation/surface reconstruction methods

All datasets were first processed using the (A) FreeSurfer v6.0^8^ reconstruction pipeline following the Desikan Killiany (DK) protocol for atlas parcellation^53^. No manual corrections were performed^54^. Given the long-standing experience with and reliability of FreeSurfer reconstructions, these were used as a silver standard comparison baseline for the human dataset despite the inherent bias towards FreeSurfer-based methods.

For reconstruction from DL-based segmentation, we focused on three different methods: (B) DeepSCAN^29^, (C) FastSurferCNN^30^ and (D) QuickNAT^28^. FastSurferCNN and QuickNAT can be regarded as variants of the same architecture, with the former being inspired by the later. All human datasets were processed by the three DL-based segmentation models. Since (D) QuickNAT cannot process skull-stripped images, it was not applied to the synthetic CTh phantom.

Previous research has confirmed that, given a high-quality segmentation of the cerebral cortex, surface reconstruction can be performed without many of the steps needed by FreeSurfer^30^. In this paper, we propose a similar fast surface pipeline; differences to^30^ are detailed in Table 2. Based on the segmentation provided by each DL model, the WM surface is reconstructed using a topology correcting fast marching algorithm^55^. This surface encloses the cerebral WM and sub-cortical structures such as the ventricles, thalamus, caudate nucleus, putamen, pallidum, hippocampus, amygdala, accumbens nucleus and ventral diencephalon. Only the pial surface is reconstructed using the FreeSurfer engines, requiring functionalities that normalize the MRI to ensure a WM signal with about 110 in gray-scale, modify the WM segmentation to avoid edges or corners and position the tessellation of the cortical surface^1^. Finally, each vertex of the tesselation is mapped on the labels provided by the segmentation with no spatial smoothing applied on the labels, allowing a region-wise analysis.

**Table 2:** Summary of the modifications made to the Fastsurfer reconstruction pipeline.

Fastsurfer processing^30^	Fast pipeline (this work)
1. DL-based brain segmentation and cortex parcellation	Yes, according to methods (B), (C) or (D)
2. Bias field correction	No
3. Linear Talairach registration	No
4. Intensity normalization	Yes
5. Corpus-Callosum segmentation	No
6. Remove edges or corners in WM segmentation	Yes
7. WM tesselation using Marching Cubes	replaced by topology correcting fast marching^55^
8. Spherical mapping	No
9. Fix topology	No
10. Place WM surface	No
11. Make pial surface (deform WM surface)	Yes
12. Map to cortical labels	Yes

All scripts necessary to run the fast pipeline reconstruction are available at https://github.com/SCAN-NRAD/DL-DiReCT-V2. The performance metrics reported in this paper were obtained using a desktop computer with 8 Intel Xeon(R) E5 1630 v3 3.70 GHz Central Processing Unit (CPU) and a 8 Gb GeForce GTX 1080 Graphical Processing Unit (GPU).

### Data analysis

From each reconstruction, we derived five standard surface-based morphometric features: the volume enclosed by the white matter (WM) and pial surfaces, their respective surface areas, and cortical thickness. Thickness estimates were computed following FreeSurfer’s definition^56^, as the average of the shortest distances from WM to pial surface and vice versa, calculated at each vertex. Hemispheric and regional comparisons were performed using the Desikan-Killiany (DK) atlas. Because FastSurferCNN outputs parcellations based on the Desikan–Killiany–Tourville (DKT) atlas^57^, regions absent from DKT (bankssts, temporal pole, frontal pole) were excluded from region-wise comparisons. Due to the small size of the affected areas these omissions had negligible impact. For the synthetic dataset, which was designed to benchmark cortical thickness estimation, we additionally compared reconstructions with DiReCT^58^, a registration-based method previously shown to detect subtle thickness changes in synthetic and real data^29,45^. An overview of all pipelines and datasets used is provided in Table 3.

**Table 3:** Summary of the processing pipelines used in each dataset. With the exception of QuickNAT, all models generate a cortex parcellation allowing the assignment of vertex-wise labels to the pial surface reconstruction.

	(A) Freesurfer		(B) DeepSCAN		(C) FastsurferCNN		(D) QuickNAT	
	Parcellation	Thickness	Parcellation	Thickness	Parcellation	Thickness	Parcellation	Thickness
ABCD	Yes	FS	Yes	FS	Yes	FS	No	FS
HCP	Yes	FS	Yes	FS	Yes	FS	No	FS
OASIS3	Yes	FS	Yes	FS	Yes	FS	No	FS
PoB	Yes	FS	Yes	FS	Yes	FS	No	FS
Synthetic	No	FS/DiReCT	No	FS/DiReCT	No	FS/DiReCT	–	–

FS, Freesurfer’s thickness; DiReCT, Diffeomorphic registration based cortical thickness measurement.

For whole-hemisphere comparisons, we performed linear regression between surface-based metrics obtained from FreeSurfer and each alternative pipeline, reporting slope and intercept with 95% confidence intervals. Regional agreement was assessed using the median percentage deviation from FreeSurfer-derived values. To evaluate scan-rescan reliability, we used repeated structural MRI scans from the Human Connectome Project (HCP), computing the root mean square (RMS) difference across all scan pairs and reporting regional averages. Although repeated scans were also available in the OASIS-3 dataset, only the HCP cohort of young healthy adults was used to avoid confounds related to image quality or advanced atrophy. To assess the influence of image contrast on reconstruction, we examined the relationship between surface-based morphometry and local white/gray matter (WM/GM) contrast in the PoB dataset. Contrast was computed following FreeSurfer conventions as $2 \times (I_{\text {WM}} - I_{\text {GM}})/(I_{\text {WM}} + I_{\text {GM}})$, where the MRI intensities $I_{\text {WM}}$ and $I_{\text {GM}}$ were sampled along the surface normal 1 mm into the WM and 30% into the cortical ribbon, respectively. For the synthetic dataset, we evaluated reconstruction accuracy using Hausdorff distance and relative errors in surface area and volume estimates. Thickness estimation accuracy was benchmarked by comparing the imposed synthetic atrophy with the values obtained from each method.

## Results

### Surface reconstruction

**Fig. 1:**
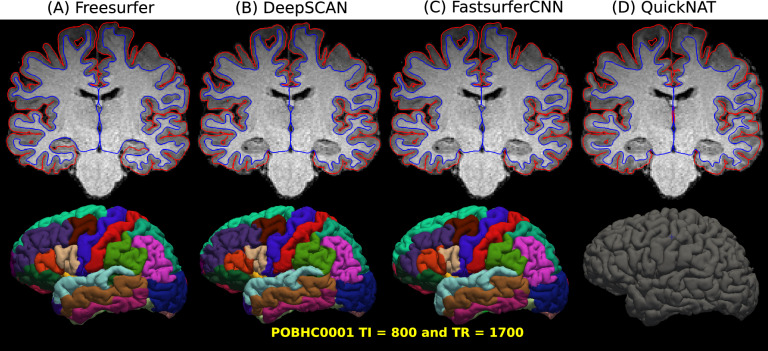
Example of the surfaces obtained from the fast surface reconstruction pipeline based on (**B**) DeepSCAN, (**C**) FastsurferCNN and (**D**) QuickNAT segmentation in comparison with the standard (**A**) Freesurfer reconstruction. The raw MRI is displayed together with the reconstructed WM (blue) and pial surfaces (red) for the same coronal slice to facilitate a direct comparison between surfaces. Apart from occasional segmentation failures, the surfaces are similar. A systematic exception is the hippocampus, which is not enclosed inside the WM for (**A**) Freesurfer. The 3D reconstruction of the pial surface showing the region labeling provided by each DL-based segmentation model allows a neuro-anatomical comparison with the DK Atlas. The subject from the PoB dataset is identified for reproducibility and the anatomical visualization was kept consistent to facilitate comparison between reconstructions. Coloring and nomenclature of brain regions adheres to^53^.

A general overview of the surfaces obtained by the fast pipeline and the cortical parcellation provided by its respective DL-based model is given in Fig. 1 using a scan from the PoB dataset with a mid-value contrast as an example. Further examples can be found in supplementary material A1.

The runtime required to reconstruct structural MRIs of healthy young adults from the HCP dataset varied by an order of magnitude at each step: from FreeSurfer (9.3 ± 2.8 h), to FastSurfer (1.7 ± 0.9 h), and further to DeepSCAN (0.18 ± 0.02 h) (see supplementary Fig. A2). Our fast surface pipeline is an adaptation of FastSurfer, designed to output only the essential elements such as cortical surfaces, vertex-wise thickness, curvature, and anatomical annotation, while omitting the many additional metrics generated by FreeSurfer and FastSurfer, which are often ignored in clinical setting due to time constraints. By focusing on minimal but interpretable outputs, our pipeline achieves a substantial runtime reduction while retaining the key features needed for future clinical applications and surface reconstruction that can be further manipulated according to the desired application.

**Fig. 2:**
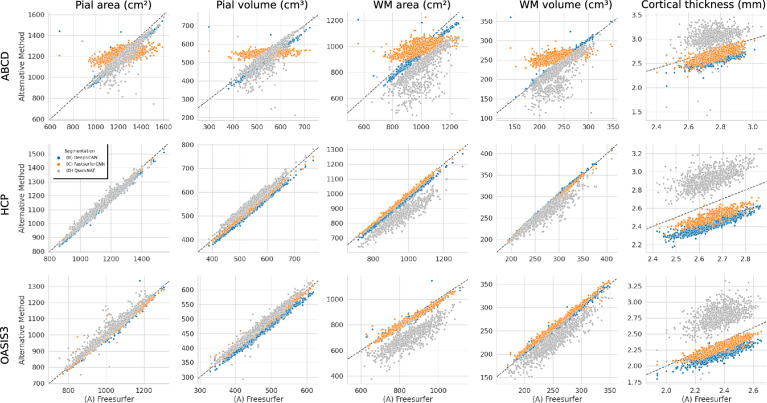
Comparison between the SBM metrics on the level of entire brain hemispheres obtained using the fast surface reconstruction based on (**B**) DeepSCAN, (**C**) FastSurferCNN and (**D**) QuickNAT segmentation models (y axes) and the standard (**A**) FreeSurfer reconstruction (always on the x axis). Each data point represents the average between the left and the right hemisphere of a subject. The identity mapping is indicated by a dotted diagonal line.

The analysis per hemisphere for subjects of the ABCD, HCP and OASIS3 datasets is shown in Fig. 2. In the B, the supplementary Table 5 summarizes the values of the slope and offset with 95% C.I. obtained by the linear regression between surface-based metrics based on FreeSurfer and the alternative fast surface reconstruction method.

**Fig. 3:**
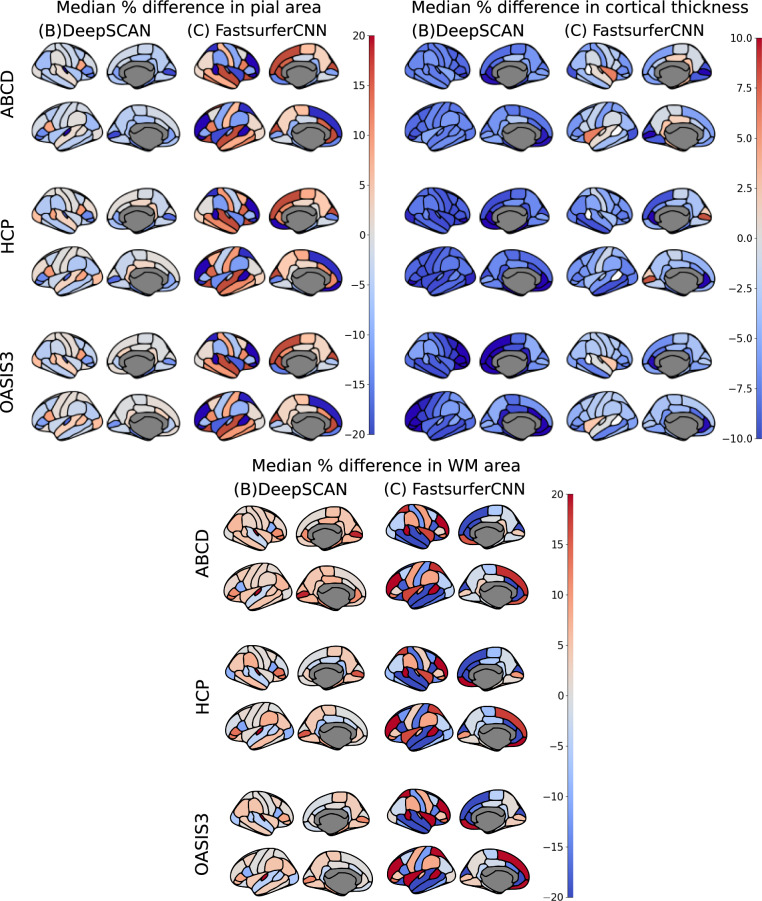
Median relative difference in the regional pial area, WM area and mean cortical thickness between Freesurfer reconstruction and the alternative fast surface reconstruction method based on (**B**) DeepSCAN and (**C**) FastsurferCNN segmentation. Negative values (blue hues) imply that values were smaller than for Freesurfer. The DK atlas was used for parcellation.

**Fig. 4:**
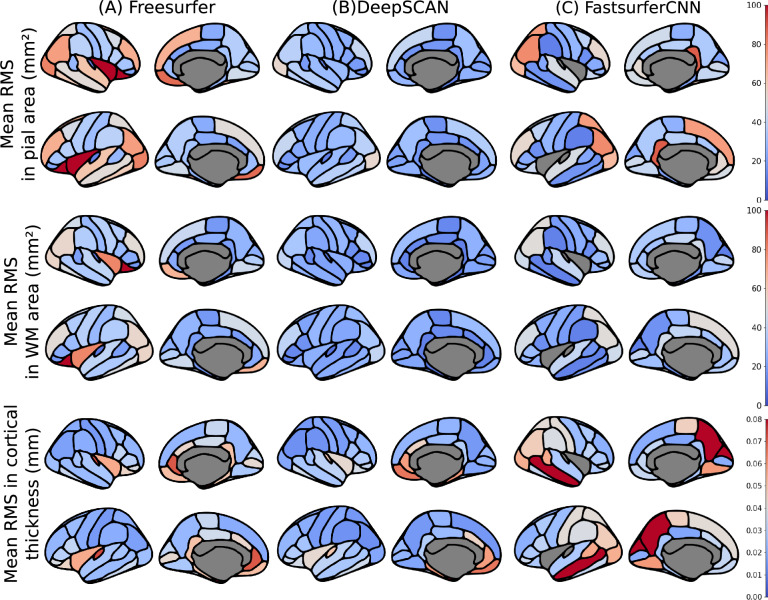
Regional scan-rescan reliability assessment of the pial surface area (top), WM surface area (middle) and cortical thickness (bottom) using HCP subjects with same-session MRI rescans (DK atlas). The mean root-mean-squared deviation of each metric is shown, using all rescan pairs of the same subject for (**A**) Freesurfer reconstruction and the fast surface pipeline based on (**B**) DeepSCAN and (**C**) FastsurferCNN.

The regional median relative difference to FreeSurfer’s pial area and cortical thickness calculated region-wise is shown in Fig. 3 following the DK atlas. Since (D) QuickNAT does not provide a parcellation of the cortex, this method is excluded here. A scan-rescan reliability assessment in HCP subjects with repeated scans is shown in Fig. 4 by the mean root-mean-square (RMS) of difference in the metrics calculated using repeated MRI scans.

To ensure that our simplification of the FastSurfer pipeline does not affect the surfaces and results presented in this paper, the full FastSurfer pipeline was used to clarify cases with large discrepancy in comparison to FreeSurfer. These results are summarized in the supplementary material C.

The impact of different grey-white contrast of the MRI on the reconstructed surface and the derived surface-based metrics is shown in Table 4, summarizing the correlation coefficient and their statistical significances for the PoB dataset, also see supplementary Fig. D1. In addition to the surface areas and the cortical thicknesses also the volumes enclosed by the pial and WM surfaces were assessed.

**Table 4:** The Pearson correlation coefficient between the surface-based metrics and WM/GM contrast. The asterisk symbol indicates statistically significant results with $p < 0.01$.

	Subject 1: 46 years			
Metric	(A) Freesurfer	(B) DeepSCAN	(C) FastsurferCNN	(D) QuickNAT
Pial area	$0.61^{*}$	0.43	$0.75^{*}$	$0.79^{*}$
Pial volume	$-0.72^{*}$	$-0.50$	$-0.90^{*}$	$-0.81^{*}$
WM area	0.08	$-0.68^{*}$	$-0.46$	$-0.73^{*}$
WM volume	$-0.72^{*}$	$-0.71^{*}$	$-0.93^{*}$	$-0.91^{*}$
Cortical thickness	$-0.54$	0.41	$-0.73^{*}$	$0.77^{*}$

	Subject 2: 38 years			
	(A) Freesurfer	(B) DeepSCAN	(C) FastsurferCNN	(D) QuickNAT
Pial area	0.35	0.26	$0.88^{*}$	$0.94^{*}$
Pial volume	$-0.55$	$-0.39$	$-0.63^{*}$	$-0.75^{*}$
WM area	0.35	$-0.55$	$-0.21$	$-0.66^{*}$
WM volume	$-0.10$	$-0.66^{*}$	$-0.85^{*}$	$-0.90^{*}$
Cortical thickness	$-0.65^{*}$	$0.62^{*}$	$-0.15$	$0.81^{*}$

### Benchmarking reconstruction pipelines

**Fig. 5:**
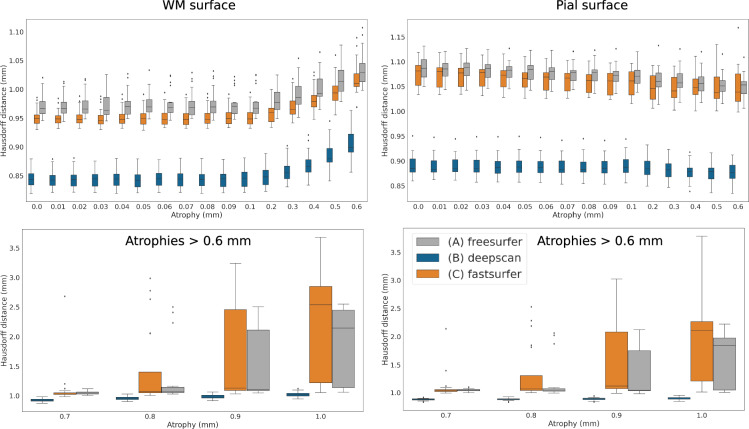
Hausdorff distance calculated between the reconstructed surface and the reference provided by the synthetic dataset. Shown is the white matter surface (left) and the pial surface (right) using the (**A**) Freesurfer reconstruction and our fast surface pipeline based on (**B**) DeepSCAN and (**C**) FastsurferCNN. For better visualization, synthetic atrophies smaller and larger than 0.7 mm are shown separately. Uniform atrophy and high atrophy levels (> 0.7 mm) are an unrealistic model of neurodegeneration. The absence of outliers in the Hausdorff distance for DeepSCAN, even for highly implausible brains, are interpreted as an evidence of the robustness of the reconstruction based on this segmentation model.

**Fig. 6:**
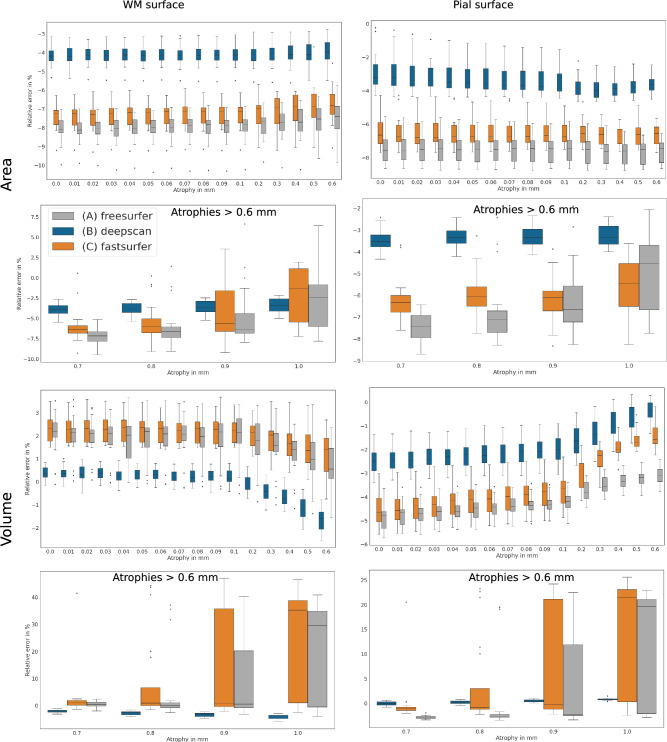
Relative error of the surface areas (top) and enclosed volumes (bottom) derived from the surfaces reconstructed by (**A**) Freesurfer and the fast surface pipeline based on (**B**) DeepSCAN, (**C**) FastsurferCNN, using the synthetic ground-truth surface as reference. Negative values represents underestimated metrics. Uniform atrophy and high atrophy levels (> 0.7 mm) are an unrealistic model of neurodegeneration. The fact that DeepSCAN based reconstructions provide the lowest values of relative error, even for highly implausible brains, are interpreted as an evidence of the robustness of these reconstructions.

**Fig. 7:**
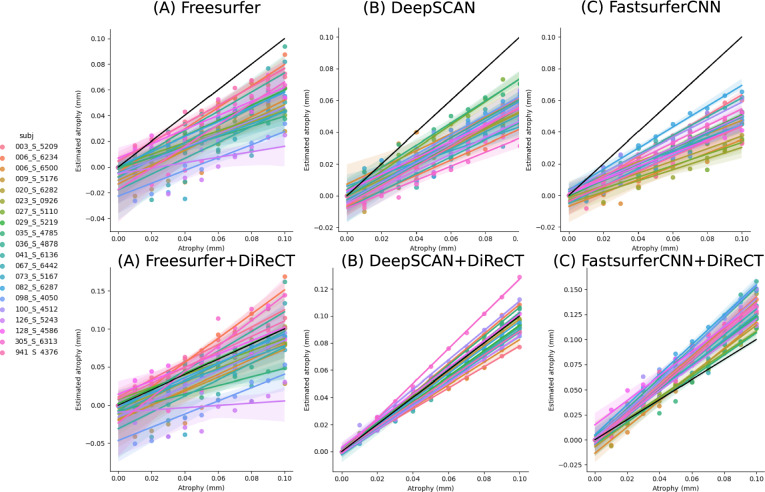
Estimated vs. induced atrophy in the synthetic dataset. For thickness estimation, both definitions, Freesurfer (top)^56^ and DiReCT (bottom)^45^ were used. The black line represents the identity.

The synthetic dataset^45^ provides an expected value for the cortical thickness and reference surfaces for the pial and white matter. Figure 5 shows the Hausdorff distance between the reconstructed surface and the synthetic ground-truth. The relative error obtained in the respective morphological variable is shown in Fig. 6. The level of atrophy estimated by each reconstruction pipeline, including measurement with DiReCT instead of FreeSurfer, is presented in Fig. 7. For all segmentation methods studied, the FreeSurfer definition of cortical thickness fails to reproduce the synthetic atrophy. In contrast, the DiReCT methodology improves the atrophy estimation showing a better performance when combined with DeepSCAN segmentation.

## Discussion

This study explored the performance of a processing pipeline to reconstruct surface meshes from structural MRI. The combination of high quality deep-learning based whole-brain segmentation with a surface reconstruction algorithm adapted from FreeSurfer renders an 11 min reconstruction of the WM and pial surfaces suitable for research applications with high-throughput requirements. This is a necessary and enabling first step toward subsequent studies aimed at evaluating robustness, pathological variability, and utility in appropriate clinical contexts, where processing speed and reliability in the individual metrics will be essential. Our approach extended previous work done in^30^ by adding the DeepSCAN model^29^), simplifying the surface reconstruction pipeline and expanding all comparisons from the voxel-based to the surface-based level. Our comparative evaluation of three deep learning models allowed benchmarking of each pipeline according to: (i) closeness to a full FreeSurfer recon-all reconstruction; (ii) reproducibility within same-session rescans; (iii) performance stability across a wide age range; (iv) sensitivity to variations of the grey-white contrast in the MRI; (v) accuracy on the metrics of synthetically produced surfaces.

The surfaces obtained from the fast reconstruction pipeline are *qualitatively similar* to the ones obtained by FreeSurfer, see Fig. 1. Apart from the mesiotemporal regions no major dissimilarities compared to FreeSurfer can be detected in the (B) DeepSCAN based reconstructions. This result holds regardless the used dataset or surface. For the ABCD dataset small deviations in the WM surface become apparent for (C) FastsurferCNN. More significant differences are present for (D) QuickNAT in all displayed reconstructions. The inaccurate surface reconstructions are caused by a systematic underestimation of the WM volume by either missing regions and/or while delimiting the WM/GM boundary. Further examples are given in the supplementary materials A and E.

*Quantitatively*, some differences between methodologies are revealed in both hemispherical and regional analysis. For the whole hemisphere, Fig. 2 and supplementary Table 5 demonstrate that the surfaces based on (B) DeepSCAN are closest to the silver-level reference provided by (A) Freesurfer for all tested datasets. This observation is confirmed by the regional analysis presented in Fig. 3, for which (B) DeepSCAN shows smaller deviations than the other models. Moreover, in the scan-rescan analysis shown in Fig. 4, (B) DeepSCAN displays a level of reproducibility higher than both (A) FreeSurfer and (C) FastSurferCNN, a result in agreement with the observation of^29^. Importantly, the surface reconstructions based on (C) FastSurferCNN fail to reproduce FreeSurfer’s result completely in the ABCD dataset. We interpret this observation as a consequence of (C) FastSurferCNN being trained on adult MRIs only. As shown in the supplementary materials C and E, these discrepancies are due to a systematic underestimation of the WM/GM boundary. Since they are maintained for the full FastSurfer reconstruction, this discrepancy is apparently not an effect of our reduced fast surface reconstruction pipeline of Table 2. Although (C) FastsurferCNN metrics derived from the whole hemisphere are close to the reference provided by (A) Freesurfer, there are significant regional differences. The reconstructions based on (D) QuickNAT display the most significant differences to (A) Freesurfer. In addition to its inability to process skull-stripped images, it also exhibiting a systematic underestimation of WM volume due to inaccurate WM/GM boundary delimitation in all datasets.

Structural MRI acquired for clinical purposes presents an inherent variability regarding not only subjects and MRI machines but also used sequence parameters. As one of the major challenges to overcome for reliable clinical routine applications in future, the significant correlation between the resulting surface metrics with the grey-white contrast of the used MRI sequence (see supplementary Fig. D1 and Table 4, and already noticed in previous studies^59,60^) need to be accounted for in longitudinal and cross sectional comparisons.

A major difference between the cortical thickness derived from FreeSurfer and the fast surface reconstruction for both (B) DeepSCAN and (C) FastsurferCNN is observed, see Fig. 3. This result is better delineated with the synthetic dataset. As shown in Fig. 7, FreeSurfer’s method to estimate cortical thickness from the mean of two straight line projections^56^ is unable to reproduce the introduced atrophy. In contrast, DiReCT’s thickness estimation^58^ provided results closer to the synthetic ground-truth regardless the underlying segmentation model. This is in agreement with previously published results^29,45^. Also, there is a general tendency of all reconstructions, including the one of our silver-standard FreeSurfer, to underestimate the sizes of the pial and WM surface of the synthetic ground-truth, see Figs. 5 and 6. Among the reconstructions, DeepSCAN showed a better performance in reconstructing the synthetic ground-truth, presenting about 1.5–2 times less relative error than the others in terms of pial area, pial volume and WM area and nearly zero relative error in the WM volume estimation. In contrast to the surfaces reconstructed by FreeSurfer and FastSurfer in case of large synthetic atrophies exceeding 0.7 mm (Fig. 5), the total absence of outliers in the Hausdorff distance for the surfaces reconstructed from the DeepSCAN pipeline suggests that no surface artifacts were created. It is important to mention that, even though the synthetic dataset is a powerful tool to benchmark the reconstruction methods, it must be considered only as a *model* of the evolution of the cortical atrophy in neurodegenerative diseases. Uniform atrophy and values as large as 0.5 mm or even more are rather unrealistic. However, the robustness in the reconstruction of these highly implausible brains can be interpreted as an indication for the high robustness of the pipeline itself.

In conclusion, our results suggest that, as long as a brain segmentation model is able to provide a high quality WM delimitation, the fast surface reconstruction pipeline is able to reconstruct reliable surface meshes. The methodology presented here extends to any segmentation map, opening the possibility of using updated versions of the current models, new DL-based segmentation models, specialized models for contrast enhanced sequences or specific diseases^61–63^. In particular, contrast agnostic models such as^63^ can be used to overcome the contrast dependency of the metrics. Among the three state-of-the-art segmentation models examined, the pipeline based on (B) DeepSCAN segmentation and DiReCT estimation of cortical thickness demonstrated the highest agreement with FreeSurfer in the human data and the greatest fidelity to the synthetic ground-truth atrophy level in the synthetic dataset. This high level of robustness across a highly heterogeneous test dataset and the remarkably accelerated runtime of only 11 min (compared to the order of 10 h for FreeSurfer and about 1 h for FastSurfer on the same computer), suggest our pipeline as a promising tool to approach future applications^14–17^. Taken together, our results provide a strong basis for future studies to more rigorously evaluate robustness, pathological variability, and real clinical utility.

## Supplementary Information

Supplementary Information.

## Data Availability

There is no data release in this paper. All dataset used are public. All scripts necessary to run the fast pipeline reconstruction are available at https://github.com/SCAN-NRAD/DL-DiReCT-V2. The DL-based cortical atrophy phantom generated by Ruskak et al. (2022) was obtained directly from the original authors upon request.
