# Supplementary material for: Fast surface reconstruction of human brain MRI: benchmarking deep-learning based morphometry tools
Source: Sci Rep. 2026 Jun 12;16:20350. doi: 10.1038/s41598-026-55397-w (PMC13328385; doi:10.1038/s41598-026-55397-w)
Supplement: Supplementary file 1 — Supplementary Information. [file 41598_2026_55397_MOESM1_ESM.pdf]

Supplementary Information

A General overview of the fast surface reconstruction

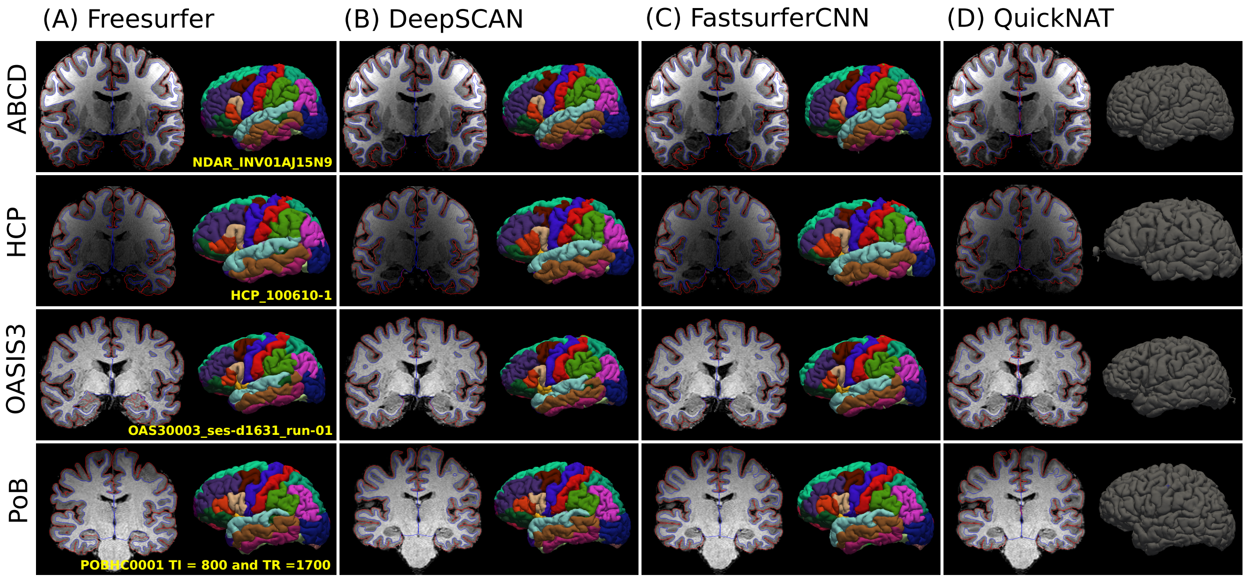

Figure A.1: Example of the results obtained from the fast surface reconstruction pipeline based on (B) DeepSCAN, (C) FastsurferCNN and (D) QuickNAT segmentation in comparison with the standard (A) Freesurfer reconstruction. The raw MRI is displayed together with the reconstructed WM (blue) and pial surfaces (red) in coronal view for the same cross-section of the brain to facilitate a direct comparison between surfaces. Apart from occasional segmentation failures, the surfaces are similar except in the hippocampus, which is not enclosed inside the WM for (A) Freesurfer. The 3D pial surface showing the region labeling provided by each DL-based segmentation model allows a neuro-anatomical comparison with the DK Atlas. The subjects are identified for reproducibility and the anatomical visualization was kept consistent to facilitate comparison between reconstructions. We retained the same coloring and naming presented in [53].

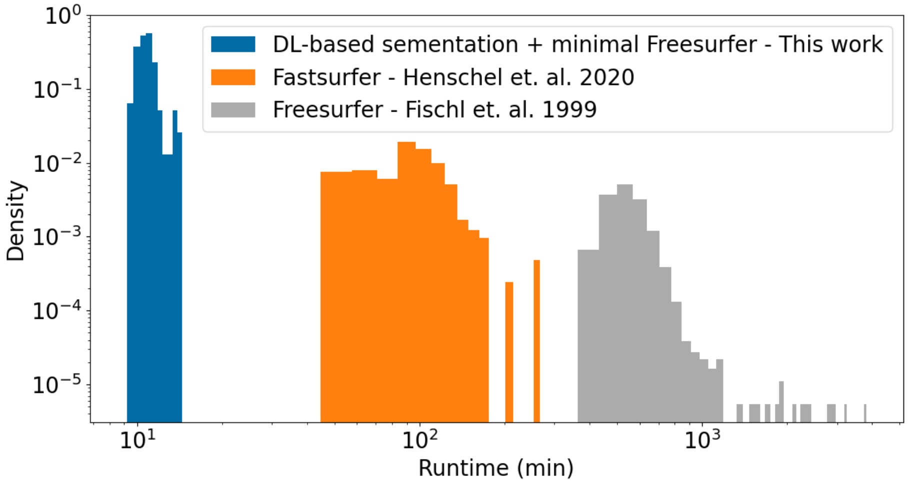

Figure A.2: The runtime distribution for the fast surface reconstruction, Fastsurfer and Freesurfer pipelines. Due to the significant difference in the time scale from minutes to hours, the results are shown on log scale.

B Table with the linear regression coefficients

Table 5: Slope and offset obtained by the linear regression between the Freesurfer's metrics and the one obtained by the alternative methods (see Figure 2).

Dataset	Metric	(B) DeepSCAN	(C) FastsurferCNN	(D) QuickNAT
ABCD	Pial area	(0.92 ± 0.01)	(0.24 ± 0.01)	(0.88 ± 0.02)
		(5 ± 1) cm^2	(91 ± 1) cm^2	(8 ± 3) cm^2
	Pial volume	(0.92 ± 0.01)	(0.07 ± 0.01)	(0.96 ± 0.04)
		(17 ± 6) cm^3	(510 ± 5) cm^3	(4 ± 200) cm^3
	WM area	(0.88 ± 0.01)	(0.25 ± 0.01)	(0.80 ± 0.03)
		(7 ± 1) cm^2	(74 ± 1) cm^2	(3 ± 4) cm^2
	WM volume	(0.96 ± 0.01)	(0.19 ± 0.01)	(0.92 ± 0.03)
		(11 ± 3) cm^3	(211 ± 3) cm^3	(8 ± 85) cm^3
	Cortical thickness	(0.80 ± 0.02)	(0.93 ± 0.03)	(0.70 ± 0.10)
		(0.35 ± 0.06) mm	(0.1 ± 0.1) mm	(0.9 ± 0.4) mm
HCP	Pial area	(0.98 ± 0.01)	(1.00 ± 0.01)	(0.95 ± 0.01)
		$-(1.3 \pm 0.3)$ cm^2	$-(0.9 \pm 0.3)$ cm^2	(5 ± 1) cm^2
	Pial volume	(0.96 ± 0.01)	(0.97 ± 0.01)	(0.92 ± 0.02)
		$-(6 \pm 1)$ cm^3	(5 ± 2) cm^3	(50 ± 6) cm^3
	WM area	(0.99 ± 0.01)	(1.00 ± 0.01)	(0.80 ± 0.01)
		$-(2.1 \pm 0.3)$ cm^2	$-(0.1 \pm 0.4)$ cm^2	(7 ± 1) cm^2
	WM volume	(1.00 ± 0.01)	(0.97 ± 0.01)	(0.80 ± 0.01)
		(2.8 ± 0.7) cm^3	(10 ± 1) cm^3	(43 ± 3) cm^3
	Cortical thickness	(0.79 ± 0.02)	(0.78 ± 0.03)	(0.78 ± 0.07)
		(0.33 ± 0.04) mm	(0.43 ± 0.08) mm	(0.81 ± 0.2) mm
OASIS3	Pial area	(0.98 ± 0.01)	(0.98 ± 0.01)	(0.99 ± 0.02)
		(0.3 ± 0.4) cm^2	(0.8 ± 0.4) cm^2	(1 ± 10) mm^2
	Pial volume	(0.94 ± 0.01)	(0.97 ± 0.01)	(1.02 ± 0.02)
		(6 ± 2) cm^3	(12 ± 2) cm^3	$-(5 \pm 5)$ cm^3
	WM area	(0.94 ± 0.01)	(0.94 ± 0.01)	(0.83 ± 0.03)
		(2.7 ± 0.4) cm^2	(3.6 ± 0.5) cm^2	(0 ± 1) cm^2
	WM volume	(0.98 ± 0.01)	(0.99 ± 0.01)	(0.91 ± 0.02)
		(7 ± 1) cm^3	(9 ± 1) cm^3	(1 ± 3) cm^3
	Cortical thickness	(0.70 ± 0.01)	(0.78 ± 0.01)	(0.82 ± 0.06)
		(0.51 ± 0.04) mm	(0.44 ± 0.04) mm	(0.8 ± 0.1) mm

C Full Fastsurfer reconstruction

The fast surface reconstructions based on (C) FastsurferCNN fail to reproduce Freesurfer in the ABCD dataset, see top row of Fig. 2. To ensure this effect is not due to our simplification of the pipeline, we have run the full Fastsurfer pipeline in a sub-sample of 100 random subjects. Figure C.1 shows that the same dissimilarity is observed in the full Fastsurfer pipeline.

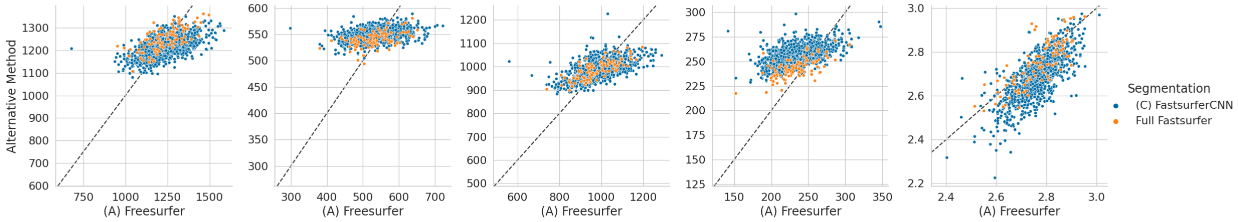

Figure C.1: Comparison between the SBM of obtained using the full FastsurferCNN pipeline and the simplified pipeline with the standard (A) Freesurfer reconstruction.

Additionally, the surface obtained by the fast methodology does not displays significant regional differences to the Full Fastsurfer as shown in Figure C.2. The median relative difference between the morphological variables derived from the full Fastsurfer and the fast reconstruction pipeline further indicates no effect on the final surface due to the pipeline simplification.

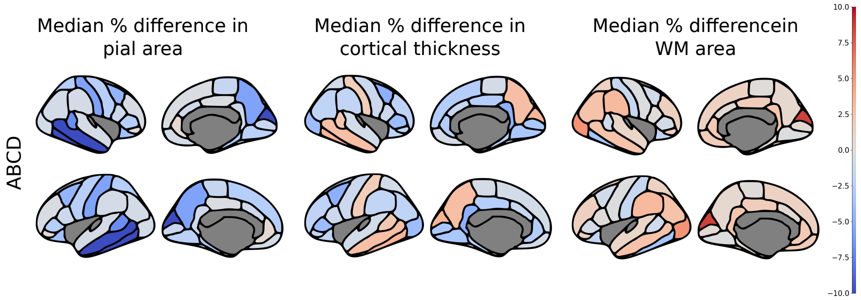

Figure C.2: The Median relative difference between the surface based morphological variables derived from the full Fastsurfer reonstruction and the fast reconstruction pipeline.

D Surface metrics and the MRI grey-white contrast

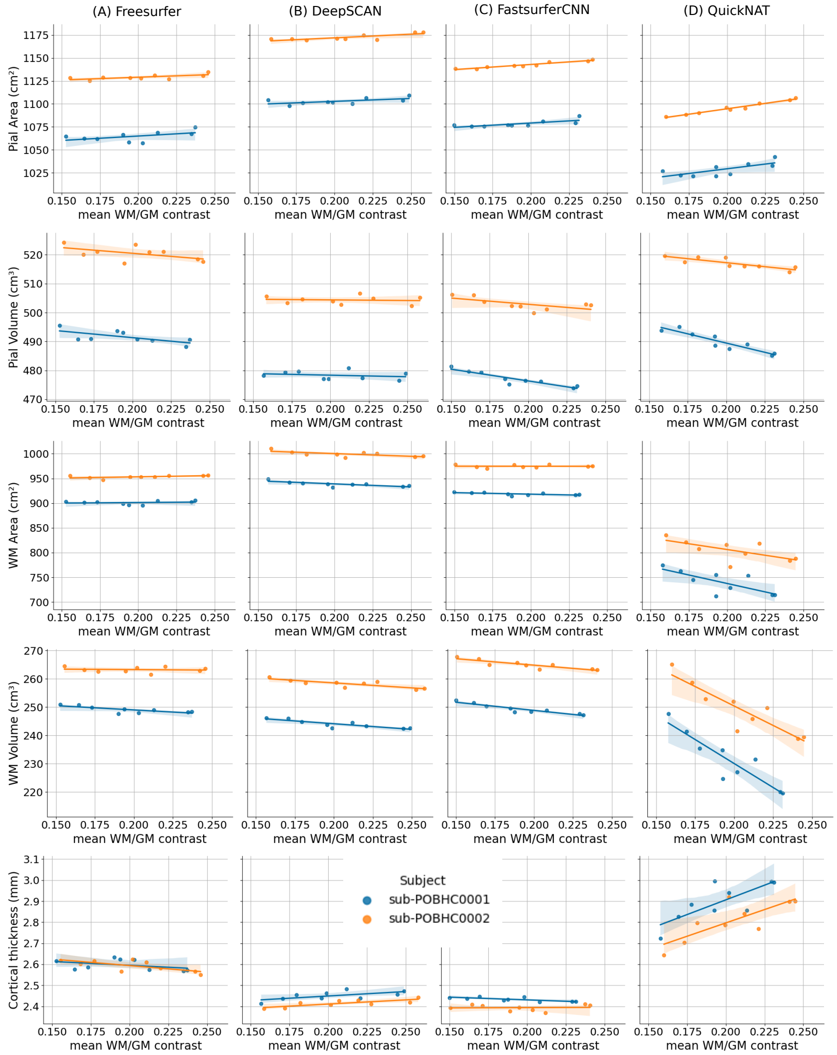

Figure D.1: Correlation between the surface reconstruction and derived morphological variables with the grey-white contrast of the MRI for (A) Freesurfer reconstruction and the fast surface pipeline based on (B) DeepSCAN, (C) FastsurferCNN and (D) QuickNAT. Each data point represents the average between the left and the right hemisphere of a scan acquired with different sequence parameters during the same session.

E General overview of the WM segmentation

As a supplement to Figure 2, a general overview of the WM segmentation provided by the three DL-based methods is shown in Figures E.1 to E.3. The representative subjects were chosen using the first (close to Freesurfer), second (median behavior) and third (far from Freesurfer) quartiles of the percentage difference between the WM volume reconstructed and Freesurfer's silver standard.

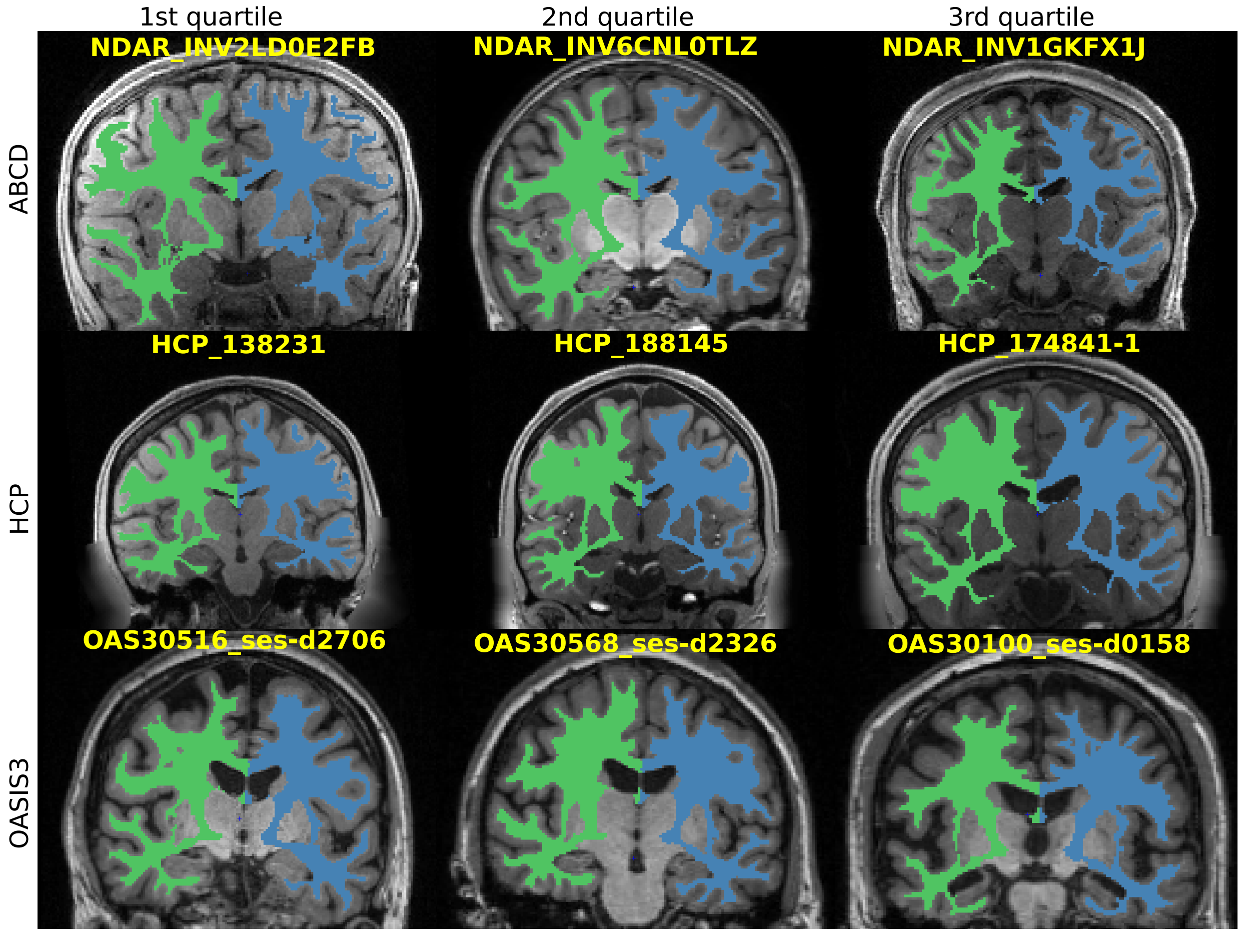

Figure E.1: Examples of the WM segmentation provided by (D) QuickNAT.

There is a systematic underestimation of the WM either missing regions and/or in the WM/GM boundary delimitation. This explains the most significant differences observed when comparing (D) QuickNAT with Freesurfer reconstruction, for all datasets. This systematic tendency to underestimate the WM volume can also be seen on (C) FastsurferCNN prediction for the ABCD dataset only. Figures E.2 and E.3 shows a general overview of the WM segmentation for both (B) DeepSCAN and (C) FastsurferCNN.

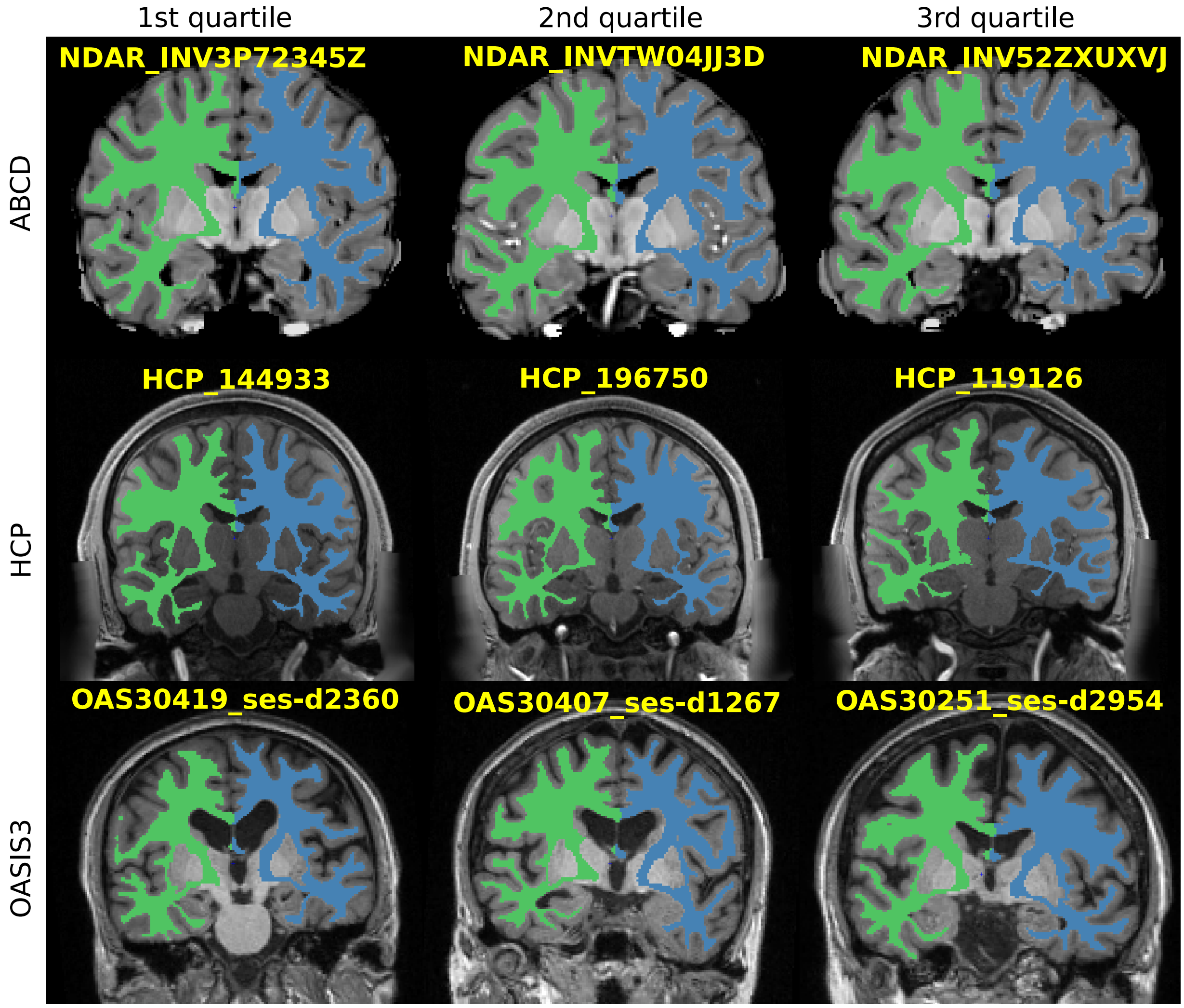

Figure E.2: Examples of the WM segmentation provided by (C) FastsurferCNN.

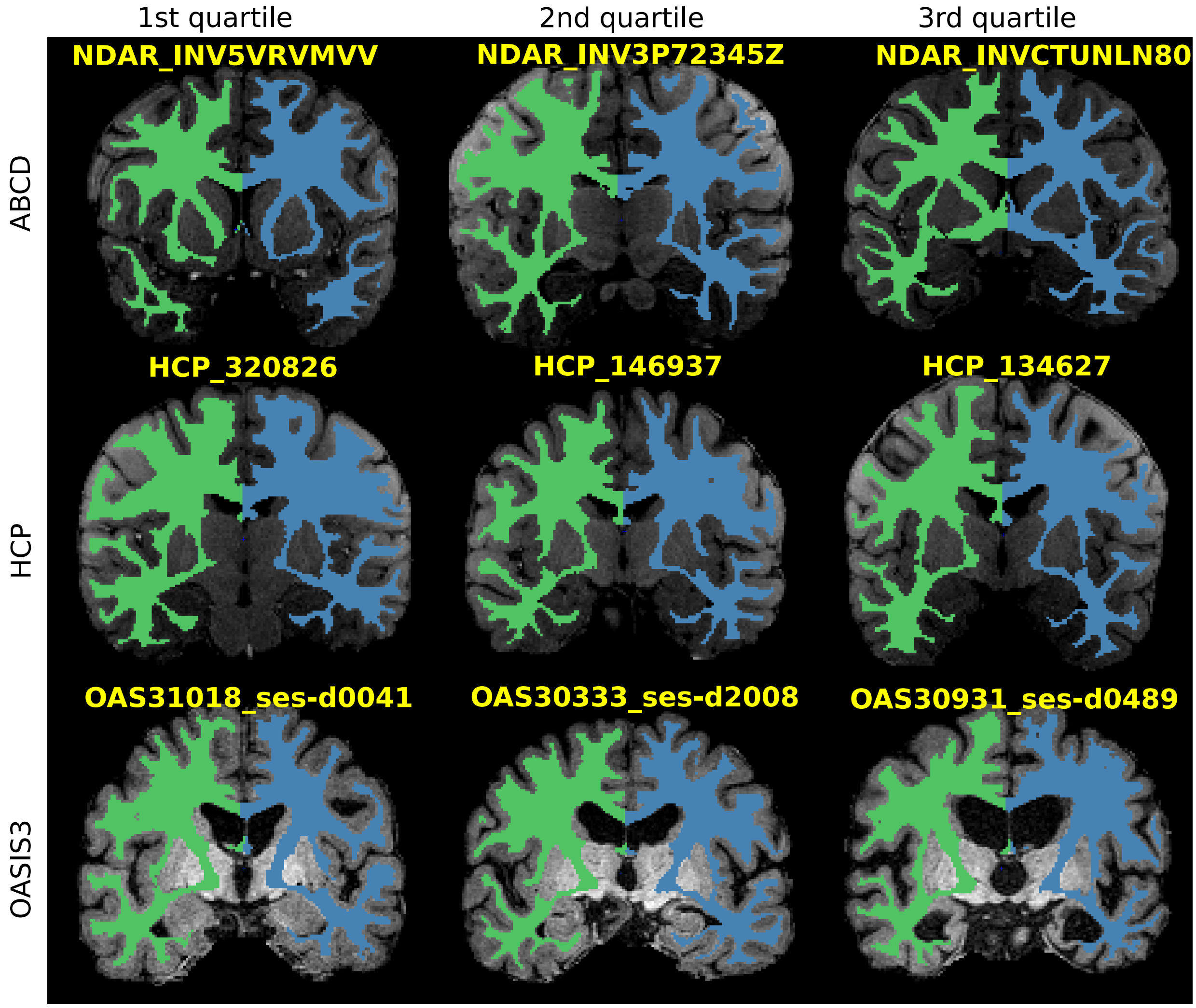

Figure E.3: Examples of the WM segmentation provided by both (B) DeepSCAN.
